# Transanal Hemorrhoidal Dearterialization (THD) Anolift-Prospective Assessment of Safety and Efficacy

**DOI:** 10.3389/fsurg.2021.704164

**Published:** 2021-09-22

**Authors:** Pasquale Giordano, Elena Schembari

**Affiliations:** Department of Colorectal Surgery, Barts Health NHS Trust, London, United Kingdom

**Keywords:** hemorrhoids, THD, Anolift, HAL, mucopexy, hemorrhoidectomy, dearterialization

## Abstract

The adjunct of a mucopexy to conventional dearterialization has become a routine part of the transanal hemorrhoidal dearterialization procedure in order to facilitate the management of the prolapsing component and has helped to expand the indications of this technique to more advanced stages of hemorrhoidal disease. A simple technical modification of THD with targeted mucopexy (TM), called Anolift, is described. The aim of the study was to evaluate the safety and effectiveness of this technical variation. The procedure consisted of two parts: one aimed at the dearterialization and the other concentrated on the management of the prolapsing component. Once all the arteries were identified and transfixed an Anolift targeted mucopexy was performed using a continuous barbed suture with a synthetic absorbable monofilament (Polydioxanone) 2/0 Filbloc (Assut Europe) stitch mounted on a 4/8 30 mm needle. Severity of hemorrhoidal symptoms was scored from 0 to 20 using a dedicated questionnaire: the Hemorrhoidal Assessment Severity Score (HASS). From May 2018 to November 2020, 60 patients with hemorrhoidal disease (HD) underwent a THD Anolift procedure. Three patients experienced severe post-operative pain and 10 (23%) suffered with difficulty in evacuation. The median follow-up period was 15.5 months (range 2–32 months). The mean HASS changed from 16.43 pre-operatively to 1.95 post-operatively (*p* < 0.0001). Pre-operative HASS very strongly correlated with the degree of hemorrhoids (*p* < 0.001), while there was no correlation between the pre-operative HASS or the degree of hemorrhoids and the post-operative HASS (*p* = 0.163). There was no significant difference in predicted post-operative HASS according to the pre-operative HD stage. One patient (1.6%) with circumferential IV hemorrhoids had a recurrence and required a further THD. Two patients had excision of skin tags (3%). The Anolift technique is safe and effective for the management of HD even in patients with advanced stages.

## Introduction

Transanal Hemorrhoidal Dearterialization (THD) has become a well-established procedure for the management of symptomatic hemorrhoids (1, 2). To facilitate the management of the prolapsing component, the adjunct of a mucopexy to conventional dearterialization has become a routine part of the THD procedure and has helped to expand the indications of this technique to more advanced stages of hemorrhoidal disease (HD) (3, 4). While the dearterialization part of the procedure is well-standardized, different techniques have been described for the mucopexy that can be performed at the same time as the dearterialization (4, 5) or can be targeted and performed separately from the dearterialization (6). In this article we present a simple technical modification of the previously described THD with targeted mucopexy (TM) (6), described as Anolift, to further optimize the management of the prolapsing component. The Anolift procedure is aimed to make the mucopexy part of the procedure easier and quicker to perform, especially when dealing with advanced stages with a very large prolapsing component, and overcome some of the pitfalls of the previous technique. The aim of the study is to evaluate the safety and effectiveness of this technical variation.

## Methods

### Study Period

May 2018 to November 2020.

### Patients

This is a prospective evaluation of all patients operated on with THD Anolift technique, as modified by the senior author. Patients with symptomatic hemorrhoidal disease of any degree that failed a conservative treatment and required surgical intervention were offered intervention in the form of THD with Anolift. Patients were not consecutive because at the beginning of the series the suture used for the Anolift was not always available, hence some patients could not be treated with this technique. All patients treated with THD Anolift were prospectively entered in a specifically designed database. Prospectively collected data included patients' demographics and relevant history. The degree of severity of hemorrhoidal symptoms was scored for each patient using a specifically designed questionnaire, the Hemorrhoidal Assessment Severity Score (HASS), assessing five different parameters, each scoring from 0 to 4 with 0 corresponding to no symptoms at all and 4 to the presence of the symptom on daily basis or with every defecation^(4)^. A total score of 0 corresponded to the complete absence of hemorrhoidal symptoms while a total score of 20 would correspond to the worst possible degree of symptoms. Patients were asked to score the worse severity of post-operative pain during the first week and on day 7 using a standardized visual analog score 0–10 (0 = no pain, 10 = the worse possible pain). The severity of the pain was defined as mild with a score from 1 to 3, moderate from 4 to 6, and severe from 7 to 10. The overall duration of post-operative pain was also recorded.

### Technique

**Figure 1:**
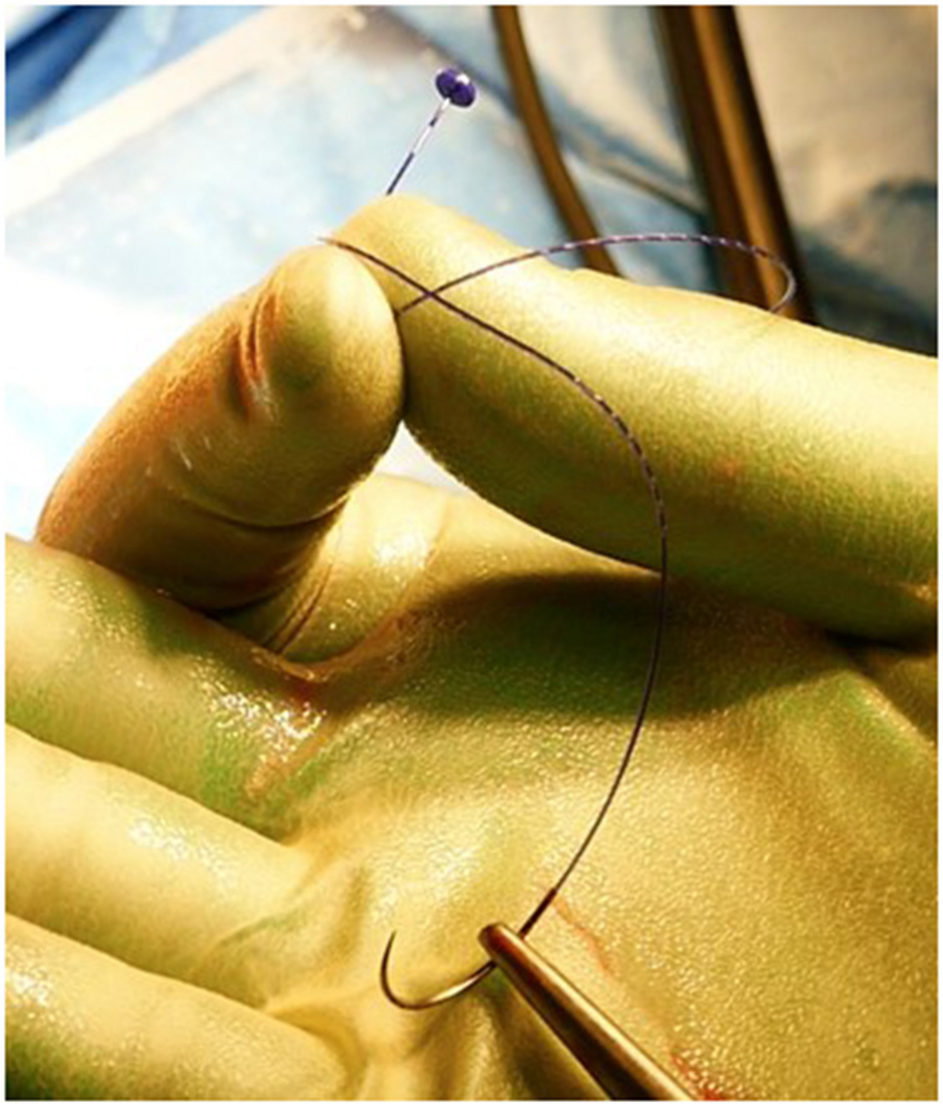
Filbloc suture.

**Figure 2:**
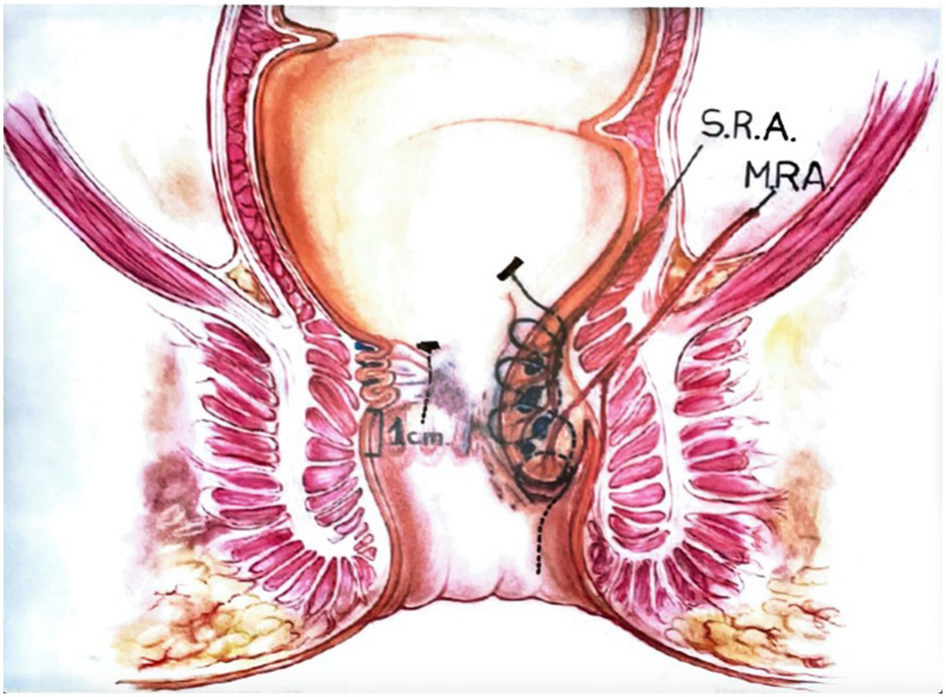
Anolift diagram.

All operations were performed as a day case under general anesthesia or alternatively under spinal anesthesia by a single surgeon (PG). Prior to surgery, a phosphate enema was administered, and a single shot of I.V. antibiotic prophylaxis was performed at induction. The procedure consisted of two parts: one aimed at the dearterialization and the other concentrated on the management of the prolapsing component. The two phases of the operation were separated so that each one of them could be optimized. The procedure was carried out in the lithotomic position using a specifically designed proctoscope (THD slide™, THD Lab™, Correggio, Italy), which incorporates a side-sensing Doppler probe and a window for placement of the sutures. The device also has a slide mechanism that, when withdrawn, allows widening of the window. The first part of the procedure consists of the dearterialization phase and was performed as previously described (4). Once all the arteries were identified and transfixed, and therefore the dearterialization phase was completed, attention was given to the areas with the largest prolapse. Once these were identified, a targeted mucopexy was performed. The aim of this part of the procedure was to correct the hemorrhoidal prolapse and any associated mucosal rectal prolapse. For this purpose, the THD slide™ was reinserted, ensuring that the instrument was introduced as deep as possible at the site of the targeted area of the prolapse. From this point, a continuous suture was started using a synthetic absorbable monofilament (Polydioxanone) 2/0 Filbloc (Assut Europe) stitch mounted on a 4/8 30 mm needle (Figure 1). This is a unidirectional barbed suture with a self-locking system. The self-locking system consists of a small button of Polydioxanone attached at the end of the suture that also serves as an anchoring point (Figure 2). The suture was progressively extended distally toward the dentate line with several passages of the needle taking the mucosa and submucosa at a distance of ~5 mm. The suture was stopped just proximal to the hemorrhoid and at least 5 mm from the dentate line, taking care not to catch the anal mucosa. During this process, the slide mechanism of the instrument was progressively widened to allow progression of the suture distally, while keeping the prolapsing mucosa away from the operating field. Once the continuous suture was completed, the sliding part was completely removed, keeping the main body of the instrument fully inserted. Once the desired level for the plication was reached, the Filbloc suture was held with one hand and was put under gentle tension while with the other hand the rectal mucosa caught by the suture was pushed up toward the proximal end of the stitch. By doing so, the rectal wall was plicated with a concertino effect and the hemorrhoid and the anal canal lifted and repositioned proximally. Any external component, if present, was not normally excised.

## Results

**Figure 3:**
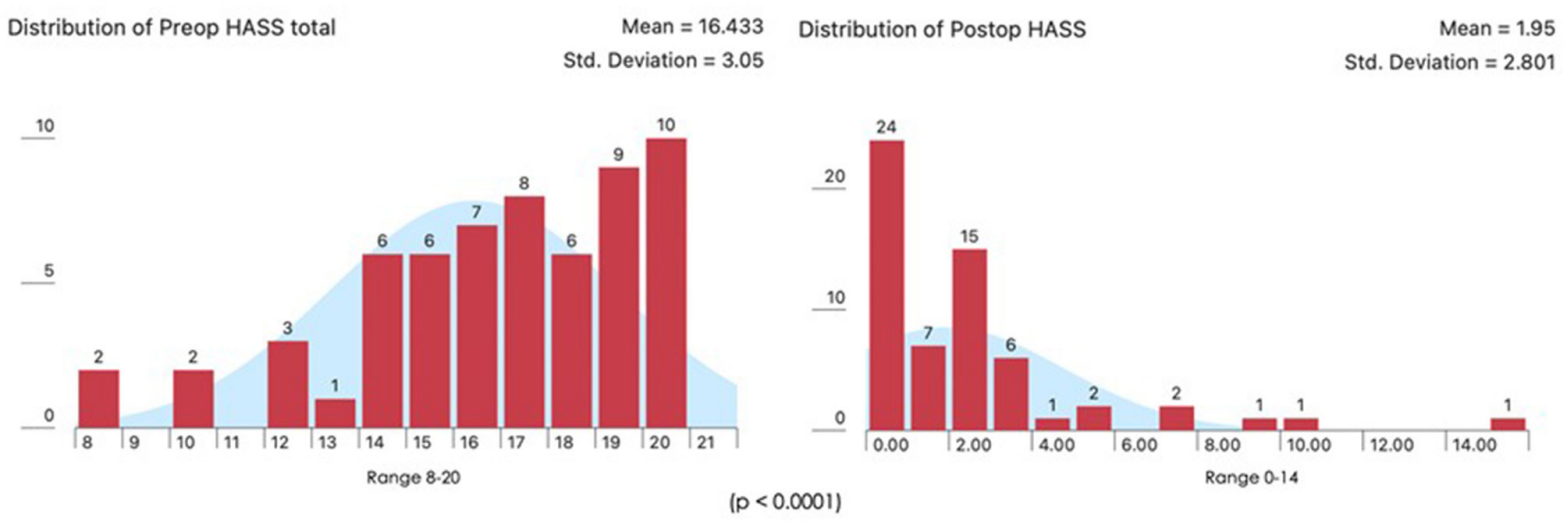
Pre-operative vs. post-operative HASS.

**Figure 4:**
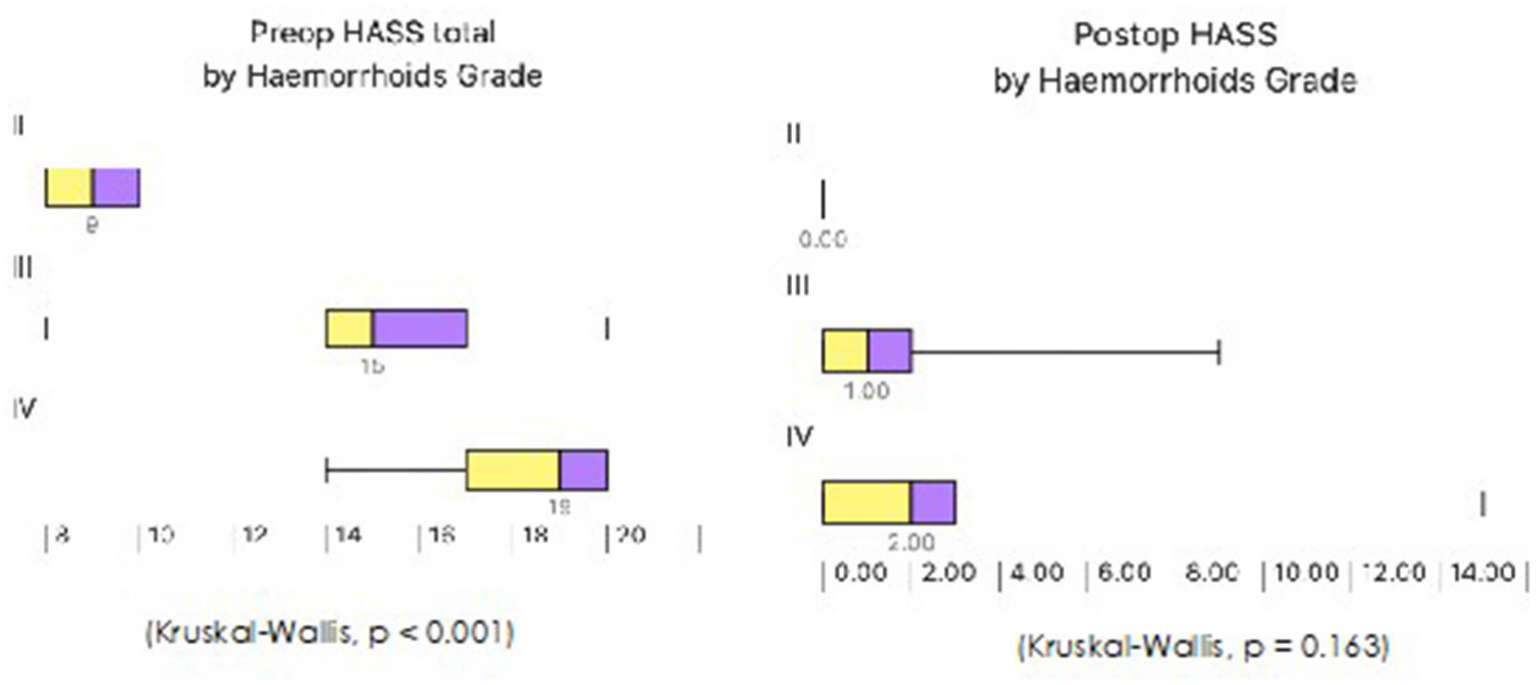
Correlation between hemorrhoids grade and HASS.

**Figure 5:**
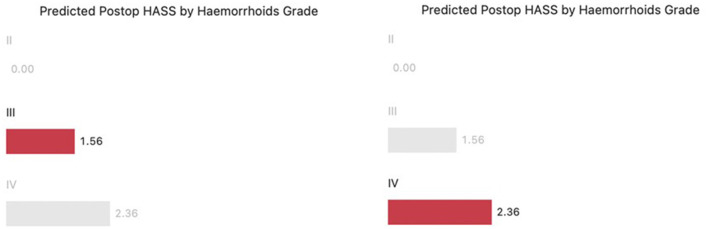
Predicted post-operative HASS by hemorrhoids grade (Kruskal-Wallis, *p* = 0.163).

A total number of 60 patients underwent THD Anolift during the study period (Table 1). The mean pre-operative HASS was 16.43 (range 8–20) (Figure 3). A six points dearterialization was performed in 55 (91.7%) patients while five arteries were identified and transfixed in the other five. A two quadrants Anolift was performed using the barbed suture in 16 (27%) patients, a three quadrants Anolift was performed in 38 (63%) patients, and a four quadrants Anolift was performed in six (10%) patients (mean of plications 2.9 ± 0.7; range 2–4). Concomitant procedures were performed in five cases (two skin tag removals, two botox injections, one open lay submucosal fistula, one single nodule haemorrhoidectomy). Post-operatively, three patients complained of a severe post-operative pain which settled within 7 days in all cases. Four patients suffered with fecal impaction (6%), and 10 (16%) reported some difficulty in evacuation which all resolved within 5 days. The median follow-up period was 15.5 months (range 2–32 months). The mean HASS changed from 16.43 pre-operatively to 1.95 post-operatively (*p* < 0.0001) (Figure 3). Pre-operative HASS very strongly correlated with the degree of hemorrhoids (Kruskal-Wallis, *p* < 0.001), while there was no correlation between the pre-operative HASS or the degree of hemorrhoids and the post-operative HASS (Kruskal-Wallis, *p* = 0.163) (Figure 4). Equally, there was no significant difference in predicted post-operative HASS according to the pre-operative HD stage (*p* = 0.163) (Figure 5). During the study period, three patients underwent further intervention. One patient (1.6%) with circumferential IV hemorrhoids had a recurrence and required a further THD Anolift to resolve the ongoing symptoms. Two patients had excision of skin tags (3%).

**Table 1:** Patients' demographics.

**Patients**	***N***	**Range/%**
**Tot**	60	
**Sex**	
Male	33	55
Female	27	
**Age (median)**	48	21–89
**Previous hemorrhoidal treatments**	11	18
Hemorrhoidectomy	4	
THD	4	
Banding	2	
Sclerotherapy	1	
**Degree**	
II	2	3
III	25	42
IV	33	55

## Discussion

A recent consensus statement on management and treatment of HD concluded that THD is a treatment option for II- and III-degree hemorrhoids and in experienced hands possibly also for IV-degree. Indeed, the scope of the mucopexy associated with the THD procedure is to correct the prolapsing component and optimize the outcome of surgery for advanced HD. This is achieved by performing a plication of the mucosa and submucosa of the rectal wall proximal to the prolapsing hemorrhoid that is lifted and repositioned in its natural position within the anal canal. The mucopexy can be performed at the same time as the dematerialization (4, 5) or can be targeted and performed separately from the dearterialization (6). A targeted mucopexy carries the advantages to perform the plication only where the prolapsing component is present using a needle and a suture different from the ones used for the dearterialization and better suited for the purpose. The 5/8 needle used for the dearterialization, while ideal for the transfixation of the artery, poses some limitations to the extent of penetration of the needle restricting the width and depth of tissue caught by the bite, making the management of large prolapsing hemorrhoids and associated mucosal rectal prolapse difficult. The use of different sizes and shapes of needle overcomes this problem. The use of a suture material like PDS also facilitates the sliding of the suture and provides a more durable support to the repair, thus optimizing the management of the prolapsing component. Finally, with the TM technique the mucopexy is only performed where the prolapsing component is most prominent, thus reducing the number of sutures needed. With this technique the length of the plication can be variable and tailored to the severity of the prolapse. For worse prolapse, the maximum length of the THD Slide™ device should be utilized. Regardless of the type of mucopexy performed, once the suture has been placed and the rectal mucosa plicated, the two ends of the suture will have to be tied. At the time of tying the knot, the proximal and distal threads of the suture are pulled together, this inevitably leading to a degree of tension at the level of the start and end point of the suture line used for the plication. The tension on the suture line will approximate these two points, causing a C deformity of the rectal wall and creating a degree of pocket effect in the rectal wall. The longer the extent of the plication the more prominent this effect may be. It is unclear whether the presence of these pockets within the rectal lumen have a detrimental effect on post-operative recovery, but it is believed that they may contribute to defecatory disturbance in the early post-operative period. Another potential concern with a conventional mucopexy in patients with a very large prolapsing component is that any excessive tension at the two extremities of the suture line may also lead to a cheese wire effect with the suture cutting through the rectal mucosa and leading to ulceration of the area and possibly counting for those very rare but significant post-operative bleeds occasionally encountered in these patients. To overcome these potential problems and simplify the procedure we have introduced a very simple variation of the previously described technique. The use of a barbed suture allows a sound and effective plication of the rectal wall with a more even distribution of the tension along the suture lines. Avoiding the need to tie the stitch eliminates the risks of creating a pocket in the rectal lumen and the possible cheese wiring effect on the rectal wall. Avoiding the knot for the plication also eliminates the risk of snapping the suture while tying the knot and may reduce operative time. The 30 mm 4/8 needle coupled with the suture fits the size of the THD device well, making it perfectively suited for the purpose, ensuring optimal room for maneuvering within the instrument. In this series of 60 patients treated with THD Anolift technique, more than half had IV-degree HD. The only post-operative events recorded were difficult evacuation in about 20% of patients and very severe post-operative pain in 5% of patients; all symptoms resolved within a week. One patient required further intervention for ongoing hemorrhoid-related symptoms. Two further patients required surgery for symptoms related to external skin tags. Including these two patients, reintervention rate was 5%. This study also demonstrated a very strong correlation between the pre-operative HASS and the degree of hemorrhoids, however, interestingly none of these two parameters correlated to the post-operative HASS, meaning that the severity of the HD did not have a negative impact on the outcome. The main limitations of this study were the fact that this was a single center study with no control group. However, this was an observational study aimed to assess the clinical outcomes of a technique that represents the evolution of a previously described technique for the treatment of hemorrhoids. This modification was introduced to overcome some of the pitfalls of the previous TM technique. Although the concept of the technique is not new and is relatively widely adopted, the way it is performed has been refined. The objective of the study was therefore to standardize the new technique and assess the safety and effectiveness of it, avoiding any potential bias. That was the reason why it was decided to start the assessment from a single center and single surgeon experience with no control group. Furthermore, we previously published a study that included 31 consecutive patients with Grade IV hemorrhoids operated on using the THD TM technique (6). Post-operative pain was reported by 22 (70%) patients on day 1 and 19 (61%) on day 7, while nine (30%) did not experience any pain at all. Overall, severe pain was reported by nine (16%) patients against 5% reported after THD Anolift. At a mean follow-up of 32 months (6–58), two (6.4%) patients required a further intervention for on-going hemorrhoidal symptoms while in the current study one out of 33 patients treated for IV-degree hemorrhoids required further intervention for residual hemorrhoidal symptoms and none in the other 27 patients. Yet, given that this was a non-comparative observational study primarily aimed to assess the safety and effectiveness of the technique, it would be challenging to assert that the Anolift modification carries any definite advantage in term of post-operative pain and recovery, or reduced morbidity and recurrence rate compared to other types of mucopexy, however, based on these preliminary results we believe all those advantages may well be added benefits of the technique.

## Conclusions

The Anolift technique is a simple and practical option that helps to simplify and optimize the management of the prolapsing component during the THD procedure. This option is safe and effective for the management of HD, even in patients with advanced stages. Based on these preliminary data it would be reasonable to call for a larger multicenter study, maybe with a control group, to further assess its role in the management of symptomatic hemorrhoids.

## Data Availability Statement

The raw data supporting the conclusions of this article will be made available by the authors, without undue reservation.

## Ethics Statement

Ethical review and approval was not required for the study on human participants in accordance with the local legislation and institutional requirements. Written informed consent for participation was not required for this study in accordance with the national legislation and the institutional requirements.

## Author Contributions

PG wrote the manuscript. ES organized the database. All the authors contributed to conception and design of the study, performed the statistical analysis, contributed to manuscript revision, read, and approved the submitted version.

## Conflict of Interest

The senior author PG is a trainer in the THD technique. The remaining author declares that the research was conducted in the absence of any commercial or financial relationships that could be construed as a potential conflict of interest.

## Publisher's Note

All claims expressed in this article are solely those of the authors and do not necessarily represent those of their affiliated organizations, or those of the publisher, the editors and the reviewers. Any product that may be evaluated in this article, or claim that may be made by its manufacturer, is not guaranteed or endorsed by the publisher.
